# The risk of central nervous system relapses in patients with peripheral T-cell lymphoma

**DOI:** 10.1371/journal.pone.0191461

**Published:** 2018-03-14

**Authors:** Dai Chihara, Michelle A. Fanale, Roberto N. Miranda, Mansoor Noorani, Jason R. Westin, Loretta J. Nastoupil, Fredrick B. Hagemeister, Luis E. Fayad, Jorge E. Romaguera, Felipe Samaniego, Francesco Turturro, Hun J. Lee, Sattva S. Neelapu, M. Alma Rodriguez, Michael Wang, Nathan H. Fowler, Richard E. Davis, L. Jeffrey Medeiros, Yasuhiro Oki

**Affiliations:** 1 Department of Lymphoma/Myeloma, The University of Texas MD Anderson Cancer Center, Houston, Texas, United States of America; 2 Department of Internal Medicine, University of New Mexico, Albuquerque, New Mexico, United States of America; 3 Department of Hematopathology, The University of Texas MD Anderson Cancer Center, Houston, Texas, United States of America; Seconda Universita degli Studi di Napoli, ITALY

## Abstract

We performed a retrospective analysis to identify risk factors and survival outcome for central nervous system (CNS) relapse of peripheral T-cell lymphoma (PTCL) by histologic type. Records of 600 PTCL patients diagnosed between 1999 and 2014 were analyzed including PTCL not otherwise specified (PTCL-NOS, 174 patients), angoimmunoblastic T-cell lymphoma (AITL, 144), ALK+anaplastic large cell lymphoma (ALCL, 74), ALK-ALCL (103), extranodal NK-cell lymphoma (ENKL, 54), or others (51). With a median follow up of 57 months, 13 patients (4 PTCL-NOS, 1 AITL, 4 ALK+ALCL, 2 ALK-ALCL, 2 ENKL) experienced CNS relapse. One-year and 5-year cumulative incidence of CNS relapse were 1.5% (95%CI: 0.7–2.8%) and 2.1% (95%CI: 1.1–3.5%), respectively. The 5-year cumulative incidence of CNS relapse was 1.8% in PTCL-NOS, 0.7% in AITL, 5.4% in ALK+ALCL, 2.1% in ALK-ALCL and 3.7% in ENKL. Extranodal involvement >1 site was the only significant factor associated with higher chance of CNS relapse (HR: 4.9, 95%CI: 1.6–15.0, p = 0.005). Patients with ALK+ALCL who had extranodal involvement >1 (N = 19) had very high risk of CNS relapse with one year cumulative incidence of 17% (95%CI: 4%-37%), all occurring within six months after diagnosis. All patients with CNS relapse eventually died (median, 1.5 months; range, 0.1–10.1 months). CNS relapse in patients with PTCL is rare event but the risk varies by subtype. ALK+ALCL patients with extranodal involvement >1 site have a very high risk of early CNS relapse, and thus evaluation of CNS involvement at the time of diagnosis and possible CNS-directed prophylaxis may be considered.

## Introduction

Peripheral T-cell lymphoma (PTCL) is a heterogeneous group of mature T-cell neoplasms that represents 5–10% of all lymphomas in the United States [1, 2]. Survival outcome of patients with PTCL remains poor, particularly in those with relapsed or refractory disease despite recent approval of novel agents [3, 4]. Central nervous system (CNS) involvement of lymphoma is an especially challenging situation. Several studies have analyzed CNS involvement and relapse in patients with PTCL and reported that risk of CNS relapse ranged from 2–6% and that survival outcome after CNS relapse was only 1–7 months [5–7]. These studies have identified some risk factors for CNS relapse in PTCL, such as elevated serum lactate dehydrogenase (LDH), extranodal involvement >1 site and high International Prognostic Index (IPI) score. However, the actual impact of histologic type of PTCL on the CNS relapse remains unclear except for that of adult T-cell lymphoma/leukemia (ATLL) associated with HTLV infection, which is reported to have high risk of CNS involvement [8], and there is no consensus regarding the indication for CNS prophylaxis. Currently, the National Comprehensive Cancer Network (NCCN) Clinical Practice Guideline only recommends CNS prophylaxis by intrathecal chemotherapy (IT) in patients with ATLL [9]. To further explore this challenging field, we performed a retrospective analysis of patients with PTCL to identify risk factors and describe survival outcome with CNS relapse.

## Patients and methods

### Patients

The study was performed in accordance with the Declaration of Helsinki and was approved by the Institutional Review Board of the University of Texas MD Anderson Cancer Center. IRB waived the requirement for informed consent due to retrospective study with chart review.

Patients with PTCL diagnosed between 1999 and 2014 were retrospectively reviewed. The diagnosis of PTCL was confirmed by hematopathologists at our center in accordance with the classification at the time of diagnosis [10–12]. Patients with CNS involvement at time of diagnosis, primary cutaneous T-cell lymphoma (CTCL), precursor T-cell lymphoblastic leukemia/lymphoma, ATLL, composite lymphoma, prior history of different lymphoma type, and concurrent diagnosis of other cancer were excluded from this study.

### Diagnosis of CNS disease

CNS (brain, spine, cerebrospinal fluid and eyes) relapse/progression of PTCL was determined based on radiological (MRI, CT head of CNS showing leptomeningeal enhancement) or pathological findings (cerebrospinal fluid cytology (CSF) showing lymphoma). Patients who showed any neurological symptoms at the time of diagnosis were evaluated for CNS involvement, but otherwise CNS specific imaging studies and/or pathologic evaluations were not performed routinely either at time of initial diagnosis or thereafter.

### Statistical analysis

Time to CNS relapse was calculated from the date of initial diagnosis of PTCL to the date of CNS relapse. We used competing risk regression analysis to calculate cumulative incidence of CNS relapse [13]. In this analysis, death without CNS relapse was defined as the competing event. Patient characteristics were analyzed for their association with time to CNS relapse using competing risk regression analysis, and the association was shown as Fine and Gray’s subhazard ratios (HR) with 95% confidence intervals (95%CI) [14].

Progression-free survival (PFS) and overall survival (OS) from time of CNS relapse was calculated from the diagnosis of CNS relapse to death from any cause using the Kaplan-Meier method. Difference in the survival function in different groups were analyzed using log-rank test.

Calculation, scoring and separation of risk groups using International Prognostic Index (IPI) and prognostic index for PTCL (PIT) were performed as previously described [15, 16]. A dummy variable was used for missing data. All analyses were performed using STATA version 13.1 (StataCorp LP, College Station, TX), with significance set at the 5% level.

## Results

### Patient characteristics

A total of 880 patients were identified in our T-cell malignancy database of which 265 patients were excluded for diagnosis described in exclusion criteria and insufficient outcome information (S1 Fig). In addition, 15 patients (8 PTCL-not otherwise specified (NOS), 3 ALK+ anaplastic large cell lymphoma (ALCL), 3 extranodal NK/T cell lymphoma (ENKL), 1 enteropathy-type T-cell lymphoma (EATL)) who had CNS involvement at initial diagnosis were also excluded, leaving a total of 600 patients for final analysis (S1 Table). The final cohort included 174 patients with PTCL-NOS, 144 angoimmunoblastic T-cell lymphoma (AITL), 74 ALK+ ALCL, 103 ALK-ALCL, 54 ENKL, 23 hepatosplenic T-cell lymphoma (HSTL), 16 EATL, and 12 subcutaneous panniculitis-like T-cell lymphoma (SPTL).

The median age of the patients was 56 years (range, 18–93 years). Seventy-six percent of patients were diagnosed with advanced stage disease, 23% of patients had performance status of 2–4, 29% of patients had bone marrow involvement, and 25% of patients had more than one site of extranodal involvement (Table 1). Sixty-three percent of patients received standard CHOP (cyclophosphamide, doxorubicin, vincristine and prednisone), 9% of patients received an etoposide containing regimen such as CHOEP or EPOCH [17], and 14% of patients received hyper-CVAD/MA or hyper-CVIDD/MA (hyper-fractionated cyclophosphamide, vincristine, dexamethasone and doxorubicin or liposomal doxorubicin alternating with methotrexate and cytarabine) [18], Among patients with ENKL, 31% of patients received an NK-cell lymphoma regimen such as DeVIC+RT (dexamethasone, etoposide, ifosfamide, and carboplatin) or SMILE (dexamethasone, methotrexate, ifosfamide, L-asparaginase, and etoposide) depending on their stage at diagnosis [19, 20], Other patients with ENKL received various chemotherapy mostly anthracycline containing, and/or radiation. CNS prophylaxis with IT chemotherapy was not routinely administered for patients with PTCL in our institution.

**Table 1:** Patient characteristics.

		All	PTCL-NOS	AITL	ALK+ALCL	ALK-ALCL	ENKL
N		600	174	144	74	103	54
Median age	(range)	56 (18–93)	56 (20–79)	63 (28–83)	32 (18–70)	57 (21–89)	52 (20–93)
Male	Male	381 (64)	114 (66)	79 (55)	47 (64)	73 (71)	35 (65)
PS	2–4	125 (23)	35 (23)	40 (31)	15 (21)	15 (17)	1 (2)
Stage	3–4	444 (76)	137 (82)	132 (95)	48 (58)	64 (64)	21 (39)
LDH	Above normal	185 (47)	60 (51)	42 (48)	24 (47)	27 (46)	13 (30)
Extranodal involvement	> 1	148 (25)	46 (26)	16 (11)	18 (25)	23 (22)	16 (30)
Bome marrow involvement	Present	167 (29)	56 (34)	59 (43)	7 (10)	14 (14)	6 (11)
IPI	High-risk (I-H, High)	159 (38)	51 (43)	42 (47)	10 (17)	19 (30)	15 (31)
PIT	High-risk (Group 3, 4)	163 (41)	47 (43)	57 (61)	9 (13)	20 (33)	9 (20)
First line chemotherapy	CHOP	379 (63)	96 (55)	107 (74)	58 (78)	74 (72)	20 (37)
	Etoposide containing regimen	52 (9)	17 (10)	18 (13)	5 (7)	6 (6)	2 (4)
	HCVAD, HCVIDD	86 (14)	37 (21)	12 (8)	3 (4)	14 (14)	5 (9)
	Others	83 (11)	24 (14)	7 (5)	8 (11)	9 (9)	27 (50)
Response to first line therapy	CR	364 (61)	88 (51)	101 (70)	58 (78)	59 (57)	34 (63)
	PR	55 (9)	26 (15)	14 (10)	2 (3)	9 (9)	2 (4)
	SD/PD	159 (27)	52 (30)	25 (17)	12 (16)	32 (34)	13 (24)

Abbreviations: PTCL-NOS, peripheral T-cell lymphoma-not otherwise specified; AITL, angioimmunoblastic T-cell lymphoma; ALCL, anaplastic large cell lymphoma; ENKL, extranodal NK cell lymphoma; PS, performance status; IPI, international prognostic index; PIT, prognostic index for PTCL-NOS; CR, complete response, PR, partial response; SD, stable disease; PD, progressive disease

### Cumulative incidence of CNS relapses and survival outcome

**Fig 1:**
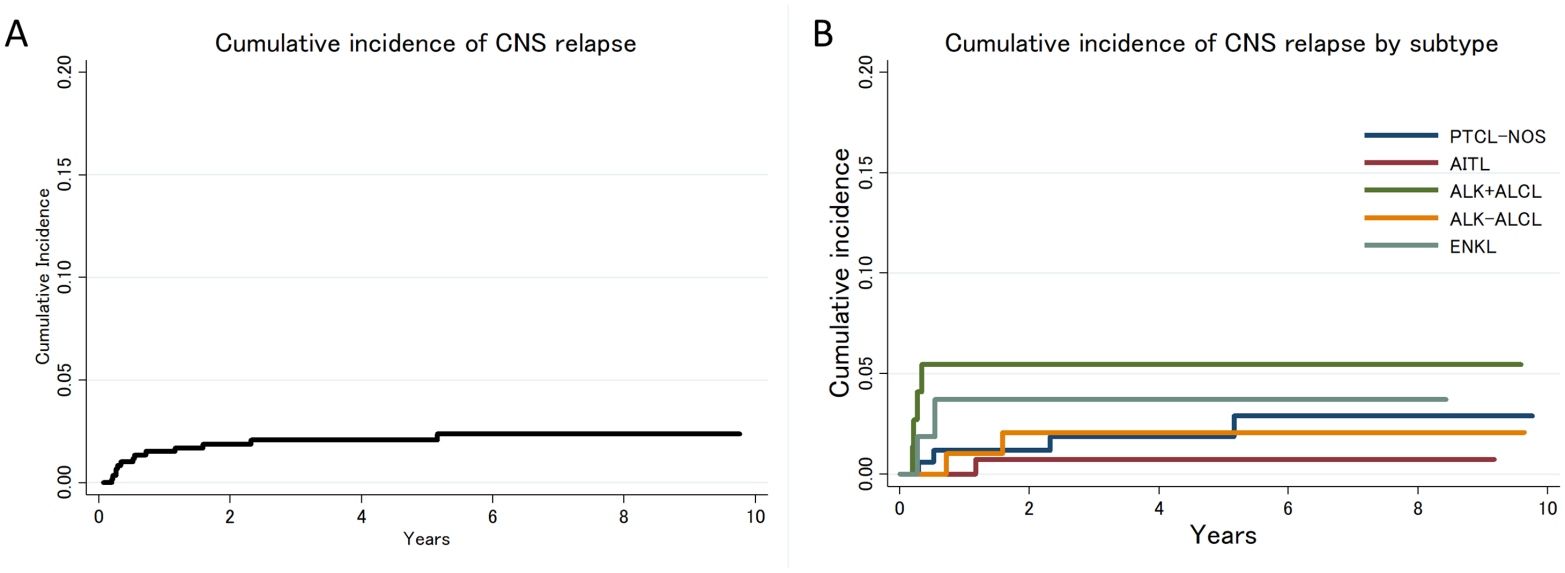
A: Cumulative incidence of CNS relapse in patients with PTCL. B: Cumulative incidence of CNS relapse in patients with PTCL by histologic types.

With a median follow up of 57 months (range: 2.3–192 months), 13 patients (4 PTCL-NOS, 1 AITL, 4 ALK+ ALCL, 2 ALK-ALCL, and 2 ENKL) experienced CNS relapse, and 309 patients (52%) died without CNS relapse. In these 13 patients, MRI brain showed leptomeningeal enhancement in 7 patients, and CSF cytology positive in 12 patients. This means that all CNS relapses were determined based on leptomeningeal involvement, and none had a form of parenchymal mass. Median time to CNS relapse was 6.4 months (range: 2.4–62.8 months). Eight patients who experienced CNS relapse (62%) did so upon first relapse/progression. One-year and 5-year cumulative incidences of CNS relapse were 1.5% (95%CI: 0.7–2.8%) and 2.1% (95%CI: 1.1–3.5%), respectively (Fig 1A). By histologic type, the 5-year cumulative incidence of CNS relapse was 1.8% (95%CI: 0.5–4.9%) in PTCL-NOS, 0.7% (95%CI: 0.1–3.6%) in AITL, 5.4% (95%CI: 1.8–12.3%) in ALK+ ALCL, 2.1% (95%CI: 0.4–6.6%) in ALK-ALCL and 3.7% (95%CI: 0.1–11.3%) in ENKL (Fig 1B). Patients with ALK+ ALCL seems to have higher incidence of CNS relapse compared with AITL, although not a statistically significant difference possibly due to the small number of cases (HR: 8.2, 95%CI: 0.9–73.5, p = 0.060).

Univariate analyses revealed that >1 extranodal site of involvement (HR: 4.9, 95%CI: 1.6–15.0, p = 0.005) is the only significant factor associated with CNS relapse in this population, which was confirmed in multivariate analyses including all available patient characteristics. No particular site of extranodal involvement was associated with a higher incidence of CNS relapse. No specific induction regimen including hyper-CVAD/MA or hyper-CVIDD/MA which involves systemic high-dose methotrexate was associated with the lower incidence of CNS relapse.

**Fig 2:**
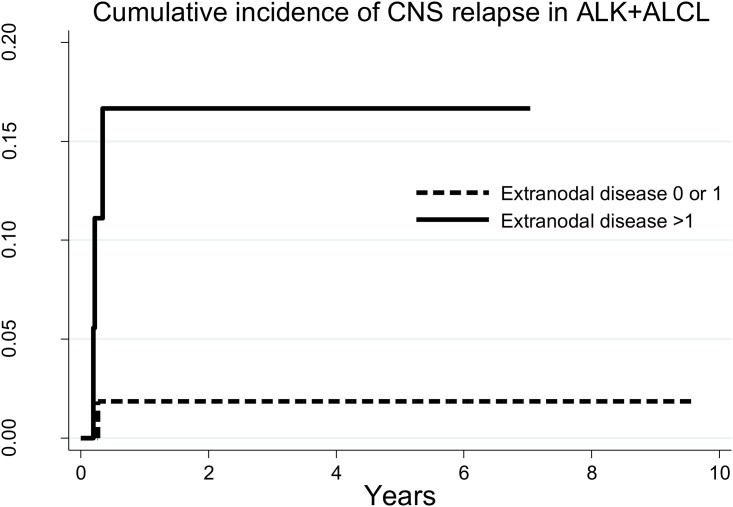
Cumulative incidence of CNS relapse in patients with ALK+ALCL by number of extranodal involvement.

Multiple sites of extranodal involvement (>1 sites) was found in 18 of 74 patients ALK+ALCL (24%). In these 18 patients, 4 experienced a CNS relapse, with one year cumulative incidence of 16.7% (95%CI: 4.1%-36.5%), all occurring within six months from initial diagnosis (Fig 2).

**Fig 3:**
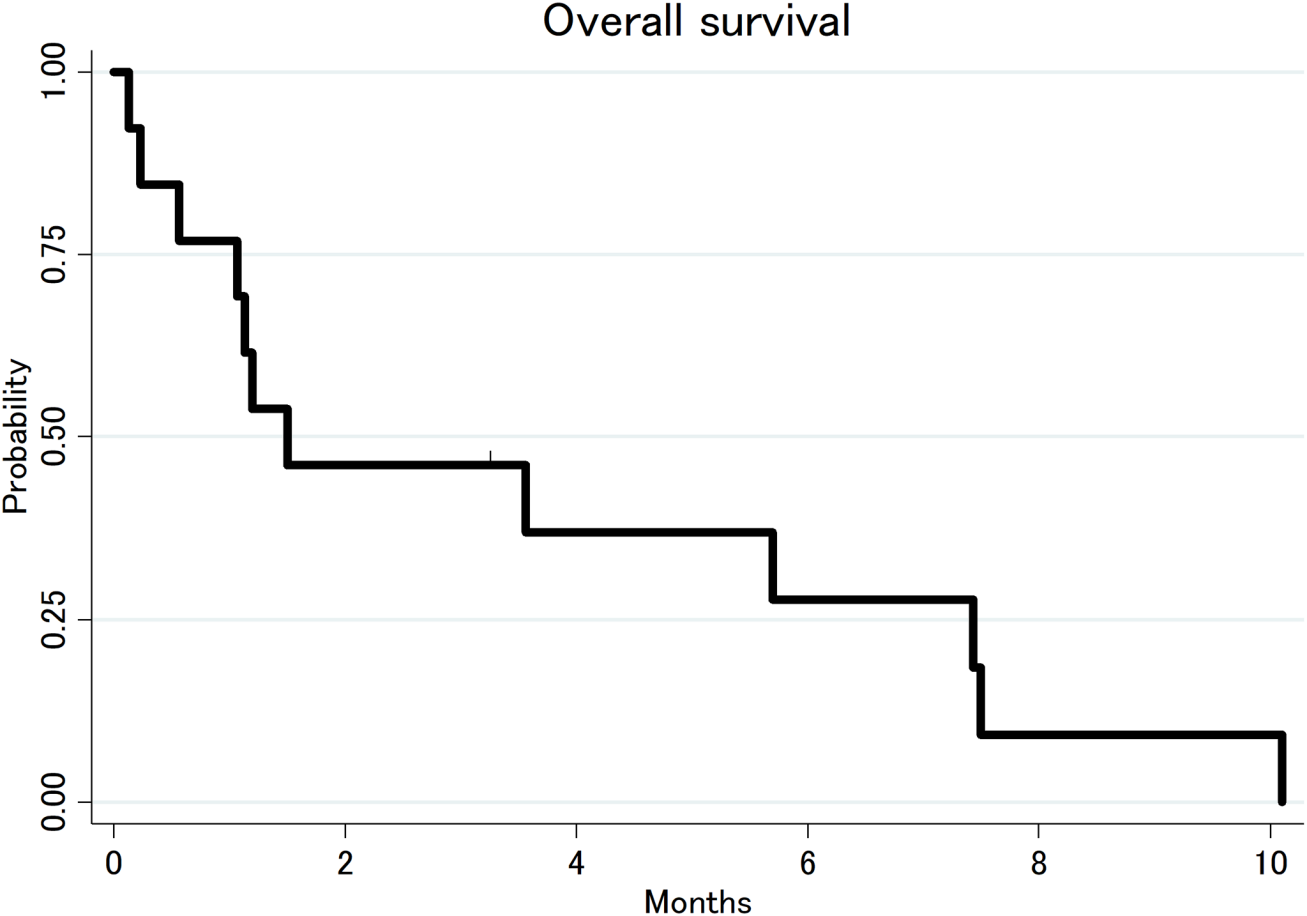
Overall survival after CNS relapse.

Four patients received best supportive care with steroid, without other chemotherapeutic treatment due to poor condition and patient’s decision, 1 received focal radiation (RT) only, 8 received salvage chemotherapy with or without IT chemotherapy. One patient underwent allogeneic stem cell transplant (allo-SCT) after achieving complete response, but died soon after transplant due to treatment complications. All patients with CNS relapse eventually died except for one patient who was lost to follow up at 2.6 months. The median OS duration from diagnosis of CNS relapse was 1.5 months (95%CI: 0.5–7.4 months) (Fig 3).

## Discussion

PTCL are heterogeneous and rare group of diseases, with various outcomes, though generally poor [21]. CNS relapse in patients with PTCL is considered a very rare event. In this study, we showed that >1 site of extranodal involvement is significantly associated with risk of CNS relapse, which is in keeping with the results from earlier studies [5–7]. Interestingly, we also found that risk may vary by histologic type; patients with ALK+ ALCL had higher risk of CNS relapse compared to other types, particularly AITL. Although multiple extranodal involvement was not necessarily a very common condition in patients with ALK+ ALCL (24%), if it was the case, such patients have a very high risk of CNS relapse with 1-year cumulative incidence of 17%. Importantly, all events for patients with ALK+ ALCL occurred within 6 months from initial diagnosis. It would be reasonable to perform a thorough evaluation for CNS involvement at the time of diagnosis in patients with ALK+ ALCL who have more than 1 extranodal site of involvement, and possibly consider CNS targeted prophylaxis.

The 5-year cumulative incidence of CNS relapse was 2.1% in our study. In a study from the Swedish Lymphoma Registry, 4.5% (28/625) of patients developed CNS relapse at a median of 4.3 months after diagnosis [6]. Yi and colleagues reported an even higher incidence of CNS involvement of 8.8% (20/228) in patients with PTCL at a median time to CNS disease of 6.1 months, though they included patients who had CNS involvement at the time of diagnosis [7]. Most recently, Gurion and colleagues presented data from Memorial Sloan Kettering Cancer Center, showing that 6.5% (15/231) of patients with PTCL had CNS involvement with a median time to CNS disease of 3.4 months, although again the authors included patients who had CNS involvement at the time of diagnosis as well as patients with ATLL; the latter group is well known to have high chance of CNS relapse [5].

The association between the site of disease involvement and future risk of CNS disease is worth evaluating. The Swedish Lymphoma Registry study identified skin and gastrointestinal tract involvement are significant risk factors for CNS relapse [6]. Yi and colleagues identified paranasal sinus involvement as a significant risk factor for CNS relapse [7]. In the current study, no specific site of extranodal involvement stood out as a risk factor. Nevertheless, having extranodal involvement in more than one site has been repeatedly shown to be a risk factor of CNS relapse regardless of sites of extranodal disease.

The role and form of CNS prophylactic treatment have been controversial [22]. Essentially all studies addressing this issue are retrospective ones. PTCL is a heterogeneous group of diseases with generally low risk for CNS relapse and whether prophylaxis should be used or not probably should be stratified by histologic types and risk factors. Patients with ALK+ ALCL have a higher risk of CNS relapse in this study making it a possible candidate for CNS prophylaxis. The study by Yi et al reported similar results although they combined ALK+ ALCL and ALK- ALCL in their analysis. In contrast, AITL is associated with a low incidence of CNS disease and CNS targeted therapy is probably not warranted.

For other types of lymphoma, studies have suggested the efficacy of systemic methotrexate for effective CNS prophylaxis [23, 24]. In this study hyper-CVAD/MA and hyper-CVIDD/MA contained a moderate dose of methotrexate, which seemed to have no significant impact on the incidence of CNS disease, and this finding is similar to an earlier study analyzing the CNS recurrence in patients with mantle cell lymphoma [25].

There are little data on the optimal treatment of CNS disease in PTCL patients. Survival outcome after CNS relapse was very poor with median OS of 1.5 months, consistent with data from other studies [6]. Not surprisingly, this seems to be shorter than the survival of patients who relapse without CNS disease [3, 4]. Further basic and clinical investigations are needed to develop novel agents with activity against CNS disease of T-cell lymphoma.

There are several limitations in this retrospective study. First, we lack central review of histopathologic diagnosis. There were changes in definition of histologic subtypes during the study period. Even though the patients who experienced CNS relapse were diagnosed all in WHO era except for one patient who was diagnosed with ALK-ALCL in 1999, cumulative incidence for subtypes should be interpreted with caution. Second, due to retrospective chart review back to 1999, information for CNS prophylaxis is not complete and thus we were not able to include this as a confounder in analysis and were not able to analyze if CNS prophylaxis had any impact on CNS relapse risk.

In conclusion, CNS relapse is rare in patients with PTCL. The risk of CNS relapse appears vary by histologic types; patients with ALK+ ALCL have a higher risk, particularly if patients have more than one site of extranodal involvement. Further studies are needed to prevent and treat this condition.

## Supporting information

S1 Fig Patient selection process. (TIF)

S1 Table Patient data. (XLSX)
