# Supplementary figures and images for: The risk of central nervous system relapses in patients with peripheral T-cell lymphoma
Source: PLoS One. 2018 Mar 14;13(3):e0191461. doi: 10.1371/journal.pone.0191461 (PMC5851529; doi:10.1371/journal.pone.0191461)

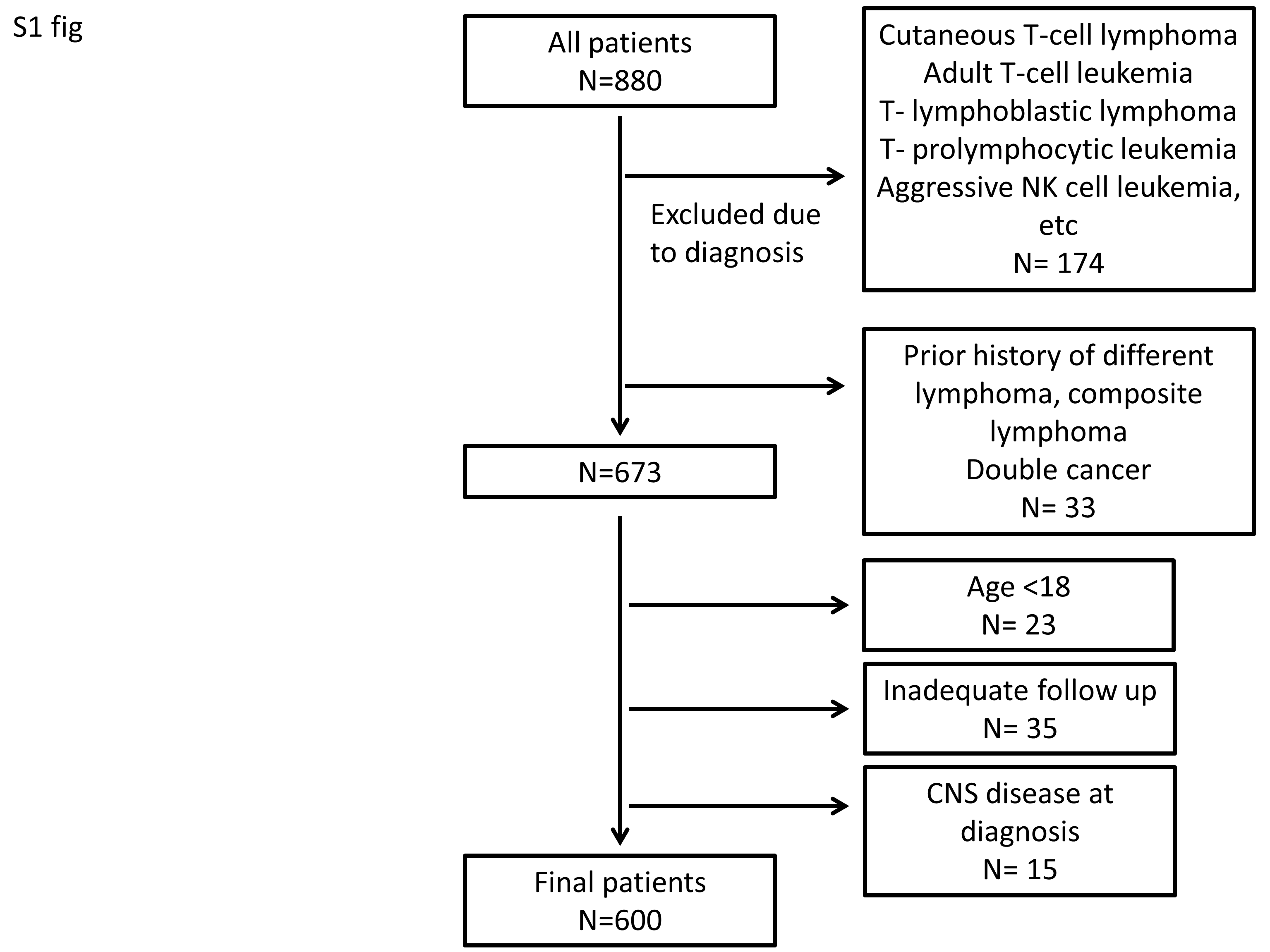

Supplement: S1 Fig — (TIF) [file pone.0191461.s001.tif]
